# Beyond National Averages: Spatial and Temporal Patterns in Type 2 Diabetes Dynamics in Romania—A 10-Year Time Series Study

**DOI:** 10.3390/clinpract16070127

**Published:** 2026-07-04

**Authors:** Cristina Gena Dascalu, Diana Tatarciuc, Magda Ecaterina Antohe

**Affiliations:** 1 Department of Medical Informatics and Biostatistics, Faculty of Medicine, “Grigore T. Popa” University of Medicine and Pharmacy, 16 Universității Street, 700115 Iasi, Romania; 2 Department of Medical Semiology, Faculty of Medicine, “Grigore T. Popa” University of Medicine and Pharmacy, 16 Universității Street, 700115 Iasi, Romania; diana.tatarciuc@umfiasi.ro; 3 Department of Implantology, Removable Dentures, Technology, Faculty of Dental Medicine, “Grigore T. Popa” University of Medicine and Pharmacy, 16 Universității Street, 700115 Iasi, Romania; magda.antohe@umfiasi.ro

**Keywords:** time series analysis, autocorrelation, cross-correlation, type 2 diabetes, epidemiology, Romania

## Abstract

Background/Objectives: This study analyzes the temporal and spatial evolution of type 2 diabetes prevalence in Romanian counties over a 10-year period (2012–2021), based on the time series autocorrelation and cross-correlation coefficients. Methods: The autocorrelation coefficients were calculated within each county’s data series, in order to identify the internal temporal dependencies, and the cross-correlation coefficients were calculated on administrative regions, in order to identify epidemiological patterns. Results: The type 2 diabetes prevalence rates in most counties follow autoregressive series, with significant positive correlations at lag 1, indicating cyclical fluctuations. Cross-correlation analysis revealed 3 regional patterns: North-East and West regions showed highly synchronized dynamics with cross-correlations exceeding 0.9, South-East and South regions showed mixed synchronization, with temporal lags of 1–2 years between counties, while the other regions (Center, South-West and North-West) revealed a synchronized core and an isolated county, with atypical dynamics (e.g., Cluj, Alba, Mehedinți, Ialomița). Conclusions: These findings highlight the utility of time series analysis in understanding the specificity of such data and their practical applicability in customizing the required public health interventions.

## 1. Introduction

In the contemporary world, Type 2 Diabetes Mellitus represents a major public health challenge, both in Romania and worldwide. This complex disease results from the interaction of genetic, behavioral, socioeconomic, and environmental factors. According to data reported in the scientific literature and based on information provided by the National Health Interview Survey, the global number of adults living with diabetes was estimated at 415 million in 2015 and is projected to increase to 642 million by 2040 [1]. The complexity of this pathology lies in the fact that Diabetes Mellitus, through its associated complications, is linked to a substantially increased risk of cardiovascular disease.

Another important factor with a profound impact on patients’ quality of life is the financial burden associated with this condition. The economic impact of diabetes was estimated at approximately 245 billion US dollars in the United States in 2012 and 1.31 trillion US dollars worldwide in 2015 [1], highlighting the considerable burden imposed on healthcare systems and society as a whole.

The distribution of Type 2 Diabetes Mellitus within the population is not uniform and is influenced by a complex combination of factors, including demographic structure, degree of urbanization, obesity prevalence, lifestyle, access to healthcare services, and the level of health education. The first Romanian study in this regard, performed at national level [2], showed that the overall prevalence of Type 2 Diabetes Mellitus adjusted for age and gender is 11.6% (95% CI 9.6–13.6%), nearly double the estimates derived from administrative registries a few years earlier, suggesting a considerable degree of underdiagnosis at the national level [3].

The prevalence of known and unknown Type 2 Diabetes Mellitus increases significantly with age, the highest prevalence being in the 60–79 years age group; there is also a higher prevalence of Type 2 Diabetes Mellitus in men than in women, whereas prediabetes is predominant in women. Overweight, general and abdominal obesity, dyslipidemia and a family history of diabetes are significantly associated with the presence of known diabetes cases. Men have a 1.5-fold higher risk to develop the disease than women, as well as the married participants (by comparison with the single or divorced individuals); the presence of hyper-LDL cholesterolemia and smoking cessation are associated with 60% lower odds of having diabetes. There are clear territorial differences concerning the prevalence, as well as the incidence of diabetes cases, ranging from below 1% to nearly 8% [3]; these differences are related to uneven population aging, disparities in obesity rates, urbanization level, dietary patterns, physical activity across regions, as well as to the access level to healthcare services, which varies considerably between urban and rural areas and between counties with different levels of economic development, influencing both diagnosis rates and disease surveillance quality [2]. In addition, vulnerable subpopulations, such as the Roma community, were identified with higher prevalence rates of diabetes and cardiometabolic risk factors compared with the general population [4], contributing therefore additionally to the territorial heterogeneity of the disease burden.

In this context, identifying and understanding these territorial differences in the evolution of Type 2 Diabetes Mellitus prevalence at the national level are essential for the development of targeted prevention strategies meant to optimize healthcare planning at both regional and national levels, by developing improved prevention programs and optimizing the allocation of healthcare resources according to the specific needs of each region.

Continuous monitoring of Type 2 Diabetes Mellitus prevalence among adults is particularly important, as recent decades have witnessed a significant increase in the number of cases, with a profound impact on patients’ quality of life. The SEARCH for Diabetes in Youth study conducted in the United States provided relevant information regarding the dynamics of this complex disease, projecting that the number of Type 2 Diabetes Mellitus cases will increase substantially as currently affected adolescents reach adulthood, from 22,820 cases in 2010 to 84,131 cases in 2050 [5].

From the combined perspective of clinical practice and public health, these trends highlight the essential role of statistical methods in quantifying the burden of disease, anticipating future healthcare needs, and supporting targeted interventions. Furthermore, identifying distinct regional patterns in the evolution of Type 2 Diabetes Mellitus prevalence may contribute to the development of prevention and management strategies tailored to local epidemiological characteristics. Such approaches may support more effective screening programs, therapeutic education initiatives, and healthcare planning, ultimately contributing to improved patient management and a reduction in the overall impact of diabetes on the population.

Time series analysis encompasses a range of statistical methods dedicated to examining and quantifying the temporal evolution of public health phenomena. The main objectives of these methods are to identify trends, seasonal patterns, cyclical fluctuations, and potential anomalies in the incidence and prevalence of diseases. Starting from a detailed description of the historical evolution of a phenomenon, particularly in the medical field, time series analysis allows for the development of statistical forecasting models that can anticipate future trends, support the understanding of potential causal relationships, and facilitate the detection of specific effects [6,7]. The information obtained through these statistical techniques is therefore essential in epidemiology, as it optimizes the implementation of prevention, education, and screening programs that are critical for disease control [8,9,10].

The investigative techniques used in time series analysis are diverse, complex, and highly valuable. From the broad spectrum of available methods, this study focuses on two specific concepts underlying the construction of valid forecasting models, which are essential in any statistical analysis of this type: autocorrelation and cross-correlation [11]. Both their theoretical aspects and practical applicability are addressed, using a real-world set of epidemiological data. Specifically, these two concepts were applied to investigate temporal and spatial similarities in the evolution of Type 2 Diabetes Mellitus cases in Romania over a 10-year period (2012–2021), both at the national level and comparatively across counties and administrative regions.

## 2. Materials and Methods

(a) *Theoretical background*: As is well known, autocorrelation, or serial correlation, measures the degree of dependency between the values of a variable observed at different points in time, indicating to what extent past values influence future values at various time lags [12]. Autocorrelation is typically denoted by ρ_k_, where k represents the lag, and is calculated similarly to the Pearson correlation coefficient; thus, for each lag k, the autocorrelation coefficient is computed as:

$$\rho_{k}=\frac{\sum_{t=k+1}^{n}\left(X_{t}-\bar{X})(X_{t-k}-\bar{X}\right)}{\sum_{t=1}^{n}{\left(X_{t}-\bar{X}\right)}^{2}},$$

where X_t_ = original data series; X_t−k_ = lagged series, and $\bar{X}$ = series’ average.

Autocorrelation is generally analyzed to identify the most appropriate time series model for forecasting. Thus, when statistically significant autocorrelations are identified, the most accurate forecasts can be generated using autoregressive models (such as ARIMA). Conversely, if no significant autocorrelations are found, this suggests that the series is stationary or follows a white noise process characterized by high random variability [13,14]. The interpretation of the autocorrelation coefficient is similar to that of the standard correlation coefficients: values close to 1 indicate strong positive autocorrelation, meaning that high past values lead to high future values in the series; values close to −1 indicate strong negative autocorrelation, where high past values correspond to low future values; and values close to 0 indicate the absence of autocorrelation at the respective lag. Values below 0.25 are considered practically null, those between 0.26 and 0.50 indicate weak autocorrelation, values between 0.51 and 0.75 indicate moderate autocorrelation, and values above 0.75 indicate strong autocorrelation (according to the general criteria formulated by Colton for the interpretation of linear correlation coefficients). It is also noted that values exceeding 0.30 already suggest a non-random time series, while values above 0.50 indicate dependencies that justify the application of autoregressive models for forecasting. When autocorrelation coefficients are calculated for multiple lags, the degree of autocorrelation at lag 1 is first examined to assess whether the time series exhibits autoregressive structure, followed by the evaluation of autocorrelations at higher lags (lags 4–5 in the present analysis), which may reveal possible trend reversals [15,16].

The identification of statistically significant autocorrelation coefficients is performed using the Box–Ljung test, which assesses whether the series exhibits overall autocorrelation across multiple lags simultaneously.

Generally speaking, cross-correlation is a statistical measure that evaluates the relationship between the movements of two datasets relative to each other [17]. It is also based on the concept of correlation, and, in the context of time series, it measures the correlation coefficients between two series by shifting them across different lags and identifying the lag at which the highest correlation occurs and the series align most closely.

In the theory of signal processing, the cross-correlation between two signals $u\left(t\right)$ and $v\left(t\right)$ is defined as $w\left(t\right)=u\left(t\right)⊗v\left(t\right)=\int_{-\infty }^{\infty }u^{*}\left(\tau -t\right)v\left(\tau \right)d\tau$, where $u^{*}\left(\tau \right)$ is the complex conjugate of $u\left(\tau \right)$ (it makes no difference if $u\left(\tau \right)$ is real-valued). In this regard, the concept of cross-correlation is quite similar to the concept of convolution between signals, considering that:

$$u\left(t\right)⊗v\left(t\right)=\int_{-\infty }^{\infty }u^{*}\left(\tau \right)v\left(\tau +t\right)d\tau \;(\mathrm{cross-correlation})\;\mathrm{and}$$

$$u\left(t\right)*v\left(t\right)=\int_{-\infty }^{\infty }u\left(\tau \right)v\left(-\tau \right)d\tau \;(\mathrm{convolution}).$$

In case of two time series X_t_ and Y_t_, the cross-correlation at lag k is calculated using the formula [18,19]:

$$\rho_{XY}(k)=\frac{\sum_{t=1}^{n}\left(X_{t}-\bar{X})(Y_{t-k}-\bar{Y}\right)}{\sqrt{\sum_{t=1}^{n}{\left(X_{t}-\bar{X}\right)}^{2}\cdot \sum_{t=1}^{n}{\left(Y_{t-k}-\bar{Y}\right)}^{2}}}$$

The lag k can be positive (when series Y is delayed relative to series X) or negative (when series Y precedes series X).

Time-series cross-correlation analysis has significant practical utility, as it evaluates the degree of synchronization between the series, allowing the determination of whether one time series influences another at a particular lag. In other words, it can help identify the impact of health-related events in one region on neighboring regions and the corresponding time delay at which this impact manifests. Cross-correlation does not demonstrate causality but may indicate potential temporal relationships and spatial synchronization patterns (e.g., specific shifts in disease burden across neighboring regions, reflecting the action of particular socio-demographic, environmental, or healthcare-related risk factors) [20,21]. The interpretation of cross-correlation coefficients is similar to that of linear correlation coefficients. Their values also range from −1 to 1, with values close to 1 indicating highly synchronized series, values close to −1 indicating series that evolve in opposite directions, and values near 0 suggesting no correlation. Values between 0.00 and 0.19 are considered practically null, values between 0.20 and 0.39 indicate weak correlation, values between 0.40 and 0.59 indicate moderate correlation and partial association between time series, values between 0.60 and 0.79 indicate strong correlation and consistent association between the series, and values between 0.80 and 1.00 indicate very strong correlation and nearly synchronized series. In addition, when interpreting cross-correlation coefficients, the lag at which the highest coefficient occurs is also evaluated: if it is 0, the series evolve perfectly synchronously; if it is positive, the second series follows the first with a certain delay (as specified by the lag); and if it is negative, the second series anticipates the first by a certain amount of time, also specified by the lag [22]. Anyway, in case of both autocorrelation, as well as cross-correlation, these categories should be regarded as descriptive guidelines meant to facilitate the interpretation, rather than as universally accepted thresholds (because they are dealing with time series, and not with regular data).

(b) *The Database*: The database used in the present study was extracted from the official report of the National Institute of Public Health—National Centre for Statistics and Health Informatics, regarding the Type 2 Diabetes Mellitus evolution in Romania during the period 2012–2021, published in 2022, publicly available on the institution’s official website (https://insp.gov.ro/download/cnsisp/Fisiere-de-pe-site-CNSISP/diabet_zaharat/Evidenta-Evolutiei-Diabetului-Zaharat-in-Perioada-2010_2021.pdf) (accessed on 23 June 2026). The data correspond to county-level registered/reported diabetes cases, collected within the national mandatory disease-reporting system, under which diabetology, nutrition, and metabolic disease units in each county are legally required to identify, notify, and maintain an active registry of diagnosed patients. No age-standardized rates were available. No missing values were present in the dataset.

Diabetes mellitus was defined according to the classical World Health Organization criteria, i.e., a fasting plasma glucose ≥126 mg/dL or a random plasma glucose ≥200 mg/dL. The data represent, therefore, administratively registered prevalence rates of diagnosed Type 2 Diabetes Mellitus cases, rather than population-based survey estimates of prevalence or incidence (such as those reported by the PREDATORR study [2]). Other newer public data regarding the Type 2 Diabetes incidence and prevalence in Romanian counties between 2022 and 2026 were unavailable at the time of this analysis—that is why the analysis is limited to the 2012–2021 interval. However, this does not jeopardize the results‘ quality, as the territorial similarities the study aims to highlight are not significantly affected by the novelty of the data, depending primarily on the accuracy and length of the investigated time series.

(c) *Methods*: A total of 43 time series were investigated (corresponding to the 41 counties of Romania, as well as to the Bucharest area and the whole country) by calculating the corresponding autocorrelations and cross-correlations.

In the first step of the study the autocorrelations for each of the analyzed time series were investigated, in order to characterize their nature. The autocorrelation coefficients were calculated using log-transformed data to stabilize variances and better capture the internal structure of the series. Considering that the time series covered a relatively short period of only 10 years, autocorrelations were calculated for lags ranging from 1 to 4, according to the Box–Jenkins recommendations to limit the maximum interpretable lag to approximately one-quarter of the series length (*n*/4) for series with fewer than 240 observations, in order to avoid unreliable estimates based on an insufficient number of paired observations [23]. The 95% confidence interval for Romania’s autocorrelation coefficient at lag 1 was estimated, using the Fisher’s r-to-z transformation, in order to identify the counties whose statistical behavior is similar to the national pattern. The autocorrelation coefficient at lag 1 was treated as the correlation between the original series and the corresponding one-year lagged series. The coefficient was first transformed as z = 0.5 × ln[(1 + r)/(1 − r)], the standard error was estimated as SE = 1/√(*n* − 3), and the confidence limits were then back-transformed to the correlation scale.

In the second stage of the study, the cross-correlations between counties were investigated. The counties were grouped according to geographical proximity, based on Romania’s eight development regions: North-East Region (Bacău, Botoșani, Iași, Neamț, Suceava, and Vaslui), South-East Region (Brăila, Buzău, Constanța, Galați, Tulcea, and Vrancea), South-Muntenia Region (Argeș, Călărași, Dâmbovița, Giurgiu, Ialomița, Prahova, and Teleorman), South-West Oltenia Region (Dolj, Gorj, Mehedinți, Olt, and Vâlcea), West Region (Arad, Caraș-Severin, Hunedoara, and Timiș), North-West Region (Bihor, Bistrița-Năsăud, Cluj, Maramureș, Satu Mare, and Sălaj), Center Region (Alba, Brașov, Covasna, Harghita, Mureș, and Sibiu), and Bucharest-Ilfov Region (Ilfov County and the city of Bucharest). This grouping method was chosen because it is based not only on geographical proximity but also on similar levels of economic development, reflected in comparable income levels and lifestyles, which may significantly influence the incidence and prevalence of type 2 diabetes cases. No formal multiple-testing correction was applied.

The calculations were made in SPSS 31.0 and Microsoft Excel.

## 3. Results

**Figure 1:**
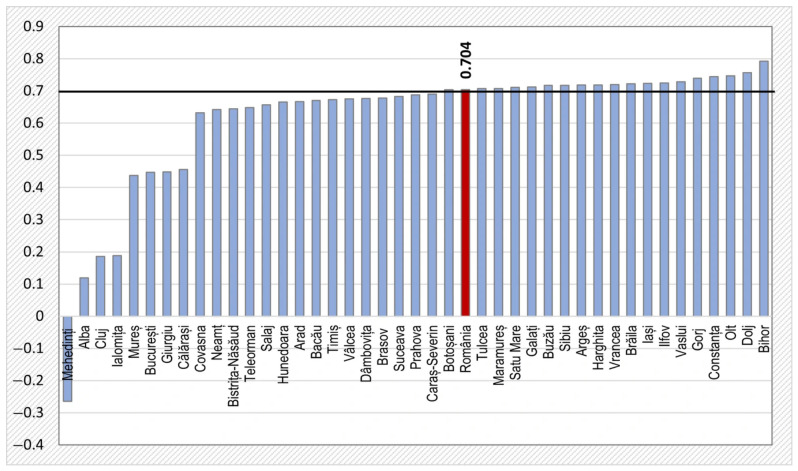
Comparative values of autocorrelation coefficients at lag 1 across all investigated time series (counties, Bucharest, and national level).

The autocorrelation coefficients calculated for the 43 investigated time-series are detailed in Appendix A and Figure 1.

It was thus observed that, in most counties as well as at the national level, there is a statistically significant, moderate-to-strong positive autocorrelation at lag 1, which gradually decreases at higher lags, indicating autoregressive time series with cyclical reversals. Several counties were identified where no dependency patterns exist between current and previous values, suggesting time series with high random variability: Alba, Cluj, and Ialomița, which showed null autocorrelations, and Călărași, Giurgiu, Mureș, Mehedinți, and Bucharest, which exhibited weak and statistically nonsignificant autocorrelations. All lag 1 autocorrelation coefficients were positive, with one exception—Mehedinți county, where a negative autocorrelation was recorded. At the national level, the lag 1 autocorrelation coefficient was 0.704; the counties displaying very similar values to the national reported value were Caraș-Severin (0.690), Botoșani (0.704), Tulcea (0.707), Maramureș (0.708), and Satu Mare (0.711). The 95% confidence interval for the national lag 1 autocorrelation coefficient was (0.134–0.924), thus encompassing almost all counties that exhibit a statistical behavior similar to the national average, with only two exceptions: Mehedinți and Alba.

**Figure 2:**
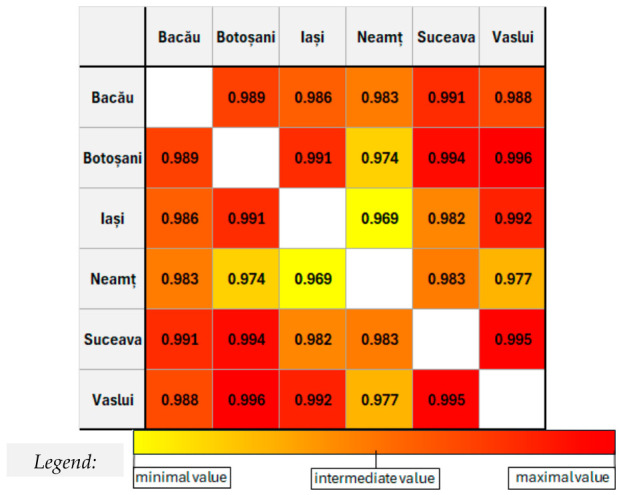
Cross-correlation coefficients at lag 0 for the counties of the North-East Region.

In the North-East Region (Bacău, Botoșani, Iași, Neamț, Suceava, and Vaslui counties), very strong cross-correlations exceeding 0.95 were detected between all counties at lag 0, indicating an almost perfect synchronization. This suggests that the prevalence of type 2 diabetes cases recorded over the 10-year study period evolved in parallel across the entire regional group, making the North-East Region a homogeneous and coherent epidemiological cluster. Additional strong correlations, indicating consistent relationships, were also observed at lags −1 and 1, highlighting inter-county delays of one year between the following pairs: Bacău and Botoșani/Iași/Vaslui; Botoșani and Iași/Neamț/Suceava/Vaslui; and Vaslui and Iași/Neamț/Suceava (Appendix B, Figure 2).

**Figure 3:**
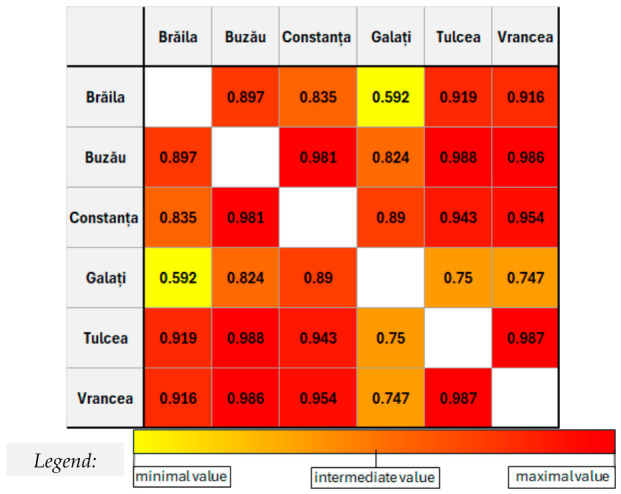
Cross-correlation coefficients at lag 0 for the counties of the South-East Region.

In the South-East Region (Brăila, Buzău, Constanța, Galați, Tulcea, and Vrancea counties), very strong cross-correlations at lag 0 were also observed between some counties, specifically Brăila–Tulcea/Vrancea/Constanța, Buzău–Tulcea/Vrancea, Constanța–Tulcea/Vrancea, and Tulcea–Vrancea. Correlations at lags 1 or −1 were slightly higher, indicating a one-year delay in the evolution of type 2 diabetes cases for certain counties. Two subgroups can thus be distinguished: a highly synchronized cluster, consisting of Buzău, Constanța, Tulcea, and Vrancea counties, and a more heterogeneous cluster formed by Brăila and Galați counties (Appendix B, Figure 3).

**Figure 4:**
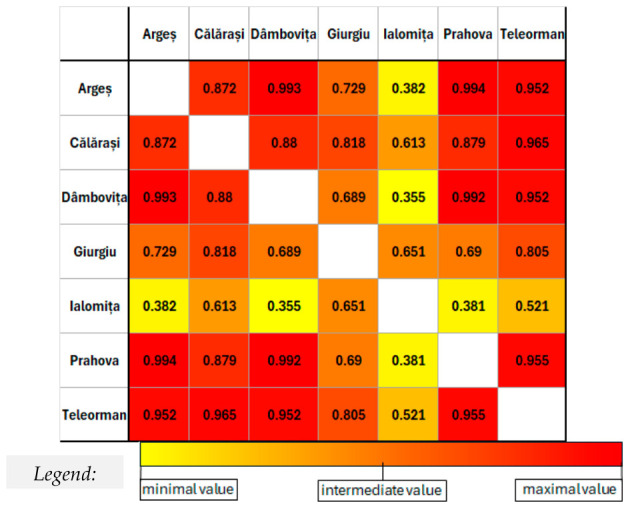
Cross-correlation coefficients at lag 0 for the counties of the South Region.

In the South-Muntenia Region (Argeș, Călărași, Dâmbovița, Giurgiu, Ialomița, Prahova, and Teleorman counties), the degree of heterogeneity increases, with greater variability observed compared to the previous regions. A group of counties still shows strong cross-correlations at lag 0, exceeding 0.9, namely: Argeș–Dâmbovița/Prahova/Teleorman, Călărași–Teleorman, Dâmbovița–Prahova/Teleorman, and Prahova–Teleorman; these counties form a well-synchronized cluster. Correlations at lags −1 and 1 are weaker, many of them being statistically nonsignificant, and some correlations were also recorded at lag −2, indicating that the inter-county delay can extend up to two years. Giurgiu and Călărași appear as a partially correlated subgroup linked to the homogeneous cluster formed by Argeș, Dâmbovița, Prahova, and Teleorman, while Ialomița County remains isolated, with weak synchronization compared to all the other counties (Appendix B, Figure 4).

**Figure 5:**
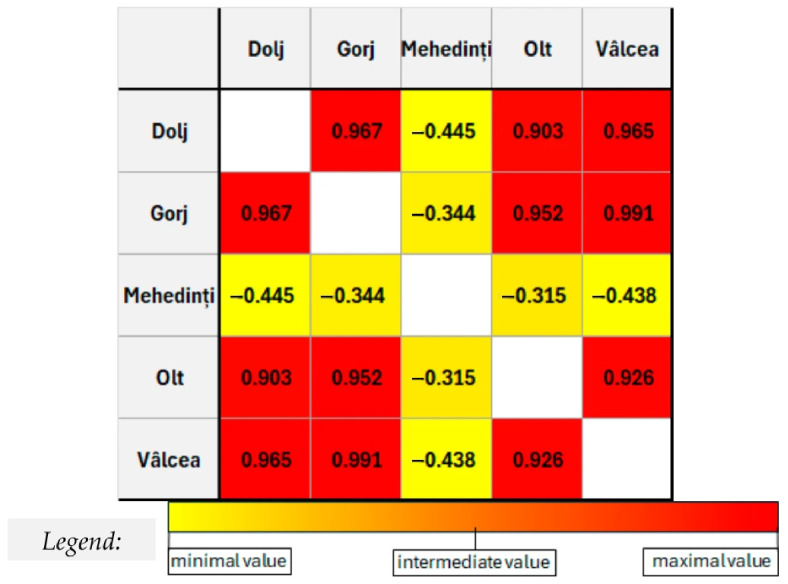
Cross-correlation coefficients at lag 0 for the counties of the South-West Region.

In the South-West-Oltenia Region (Dolj, Gorj, Mehedinți, Olt, and Vâlcea counties), a cluster of highly synchronized counties was also identified at lag 0, with cross-correlations exceeding 0.9: Dolj, Gorj, Olt, and Vâlcea. Mehedinți county stands out with an isolated behavior, displaying negative, statistically nonsignificant, and weak-to-moderate correlations with the other four counties. Strong and statistically significant correlations were also identified at lags 1 and −1, indicating an increasing one-year delay between certain counties (Appendix B, Figure 5).

**Figure 6:**
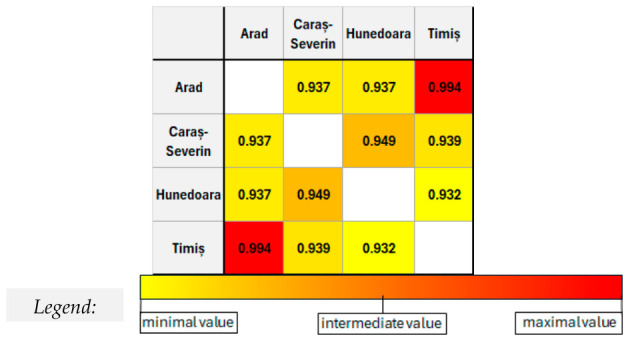
Cross-correlation coefficients at lag 0 for the counties of the West Region.

In the West Region (Arad, Caraș-Severin, Hunedoara, and Timiș counties), very strong and well-synchronized correlations were again identified between all four counties at lag 0, with the strongest correlation observed between Arad and Timiș, which were almost perfectly synchronized, with a cross-correlation coefficient of 0.994. One-year delays were also detected, but without a systematic direction and with relatively low amplitude. No peripheral counties were identified, and the degree of synchronization was comparable to the high level of homogeneity observed in the North-East (Moldova) Region (Appendix B, Figure 6).

**Figure 7:**
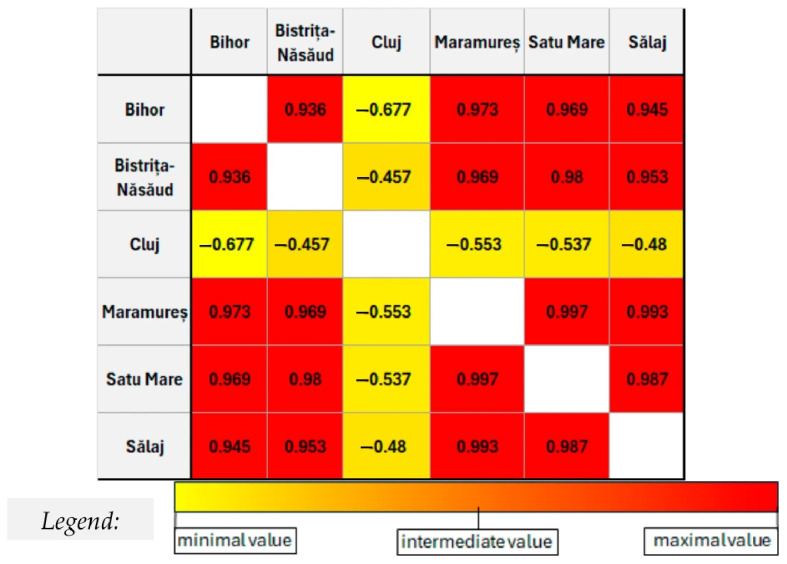
Cross-correlation coefficients at lag 0 for the counties of the North-West Region.

In the North-West Region (Bihor, Bistrița-Năsăud, Cluj, Maramureș, Satu Mare, and Sălaj counties), a cluster of highly synchronized counties was also identified, with cross-correlations exceeding 0.9—including Bihor, Bistrița-Năsăud, Maramureș, Satu Mare, and Sălaj. Cluj county stands out with a discordant pattern, showing statistically nonsignificant and negative correlations with all the other counties. Lags ±1 were also frequently observed; these delays are moderate in amplitude and do not affect the overall synchronization. The observed situation is similar to that found in the South-West Region, with a cluster of highly synchronized counties and one completely isolated county that does not follow the regional pattern (Appendix B, Figure 7).

**Figure 8:**
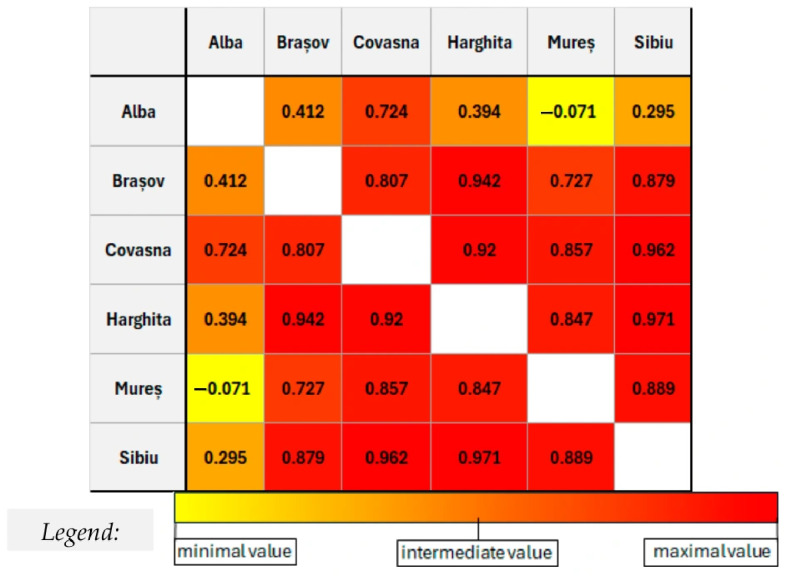
Cross-correlation coefficients at lag 0 for the counties of the Center Region.

In the Center Region (Alba, Brașov, Covasna, Harghita, Mureș, and Sibiu counties), a cluster of well-synchronized counties was again observed at lag 0, with cross-correlation coefficients exceeding 0.9: Brașov, Harghita, Sibiu, and Covasna. Mureș county showed slightly weaker correlations with this core group, indicating a consistent, but rather reduced association. Once again, one county displayed isolated behavior—Alba—which showed no correlation with Mureș, nonsignificant correlations with Brașov, Harghita, and Sibiu, and a strong correlation with Covasna. Lag ±1 correlations were again present, though moderate in amplitude, indicating only slight delays between certain county pairs (Appendix B, Figure 8).

**Figure 9:**
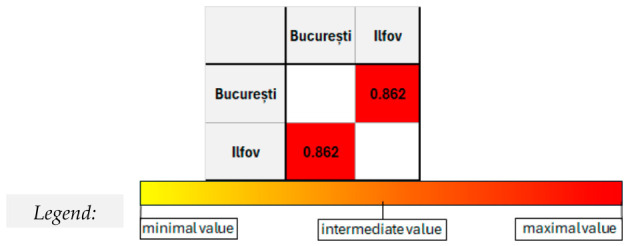
Cross-correlation coefficients at lag 0 for the counties of the București-Ilfov Region.

In the Bucharest-Ilfov Region (Ilfov county and the city of Bucharest), a very strong correlation (0.862) was observed at lag 0, indicating synchronized behavior between the two time series. The correlation at lag 1 was also relatively high, suggesting a slight delay in amplitude between the two regions (Appendix B, Figure 9).

A summary of these results, including the identified synchronization patterns, the core group of highly synchronized counties (cross-correlation at lag 0 exceeding 0.9), the atypical or isolated counties, and the predominant lag structure observed between county pairs across all eight administrative regions is presented in Table 1.

## 4. Discussion

The current study, aiming to investigate the temporal synchronization of reported Type 2 Diabetes Mellitus cases across Romanian counties over a 10-year period, highlights clear differences between the country’s administrative regions, outlining a map of the regional dynamics of this disease in Romania.

The analysis of autocorrelation coefficients at the county level indicates, in most cases and also at the national level, the presence of autoregressive time series with cyclical reversals. However, several counties exhibit specific behavior characterized by high random variability—Alba, Cluj, Ialomița, Călărași, Giurgiu, Mureș, Mehedinți, and even Bucharest—which do not follow the general trend and function as atypical areas. This characteristic is also highlighted through the analysis of cross-correlation coefficients at the level of Romania’s administrative regions.

Three general patterns were identified as characterizing the prevalence of Type 2 Diabetes Mellitus cases across Romania. The *first pattern* is represented by the almost perfect synchronization of all counties within a region, with no time delays. This pattern is observed in the North-East (Moldova) and West (Banat) regions, where cross-correlation coefficients at lag 0 exceed 0.9, indicating that the temporal fluctuations in Type 2 Diabetes Mellitus cases over the 10-year monitoring period followed nearly identical trends. The *second pattern* consists of two regional clusters: one homogeneous and highly synchronized, and the other more heterogeneous but still strongly connected to the first. This situation is found in the South-East and South regions, which are characterized by more frequent temporal variability and multiple significant correlations not only at lag 0 but also at lags −1 and +1. The *third pattern* involves the presence of a homogeneous and well-synchronized regional cluster alongside an isolated county with atypical behavior—as seen in the North-West Region with Cluj County, the Center Region with Alba County, and the South-West Region with Mehedinți County. The atypical behavior of these counties cannot be explained by taking into consideration only the statistical analysis of time-series data. It is possible instead to formulate plausible exploratory hypotheses, derived from the known socio-demographic profile of these areas, which can be further explored and formally tested in future studies. In the case of Cluj County, its accelerated economic development, high urbanization level, and academic/university profile may be associated with different lifestyle and dietary patterns, as well as more extensive access to medical services and screening programs, compared with the rest of the North-West Region; these factors could plausibly contribute to a divergent epidemiological pattern, although this remains to be formally tested. For Alba County, its transitional geographic position, lower population density, and potentially more limited healthcare infrastructure could represent alternative explanatory hypotheses. Similarly, in the case of Mehedinți County, a more pronounced degree of population aging and reduced access to healthcare services may be hypothesized as contributing factors, given the demographic profile of this region; however, such hypotheses require confirmation through further multivariate analyses incorporating such socio-demographic and healthcare access indicators.

The region with the most accentuated degree of heterogeneity was the South—Muntenia Region, which exhibits statistically the highest variability in epidemiological synchronization, with a wide dispersion of cross-correlation coefficients at lag 0, ranging from nonsignificant values (below 0.40) to values close to 0.99. Significant correlations were also identified at lags of 1–2 years, suggesting fragmented prevalence rates within the region. The plausible explanatory hypothesis for this fragmentation can be the more pronounced socio-economic diversity of the region, with substantial differences between the southern counties (e.g., Teleorman, Giurgiu, Ialomița) and the sub-Carpathian counties (e.g., Prahova, Argeș, Dâmbovița). The sub-Carpathian counties have experienced more rapid economic development and higher levels of urbanization, which directly improves the quality of available healthcare infrastructure and services, thereby increasing the population’s access to these services. By contrast, the southern counties (like Ialomița) have predominantly a rural profile, involving a specific demographic structure and potential differences in healthcare accessibility and disease reporting practices, which can justify their specific behavior. Anyway, these are again only plausible hypotheses, requiring confirmation through further analyses incorporating more specific socio-demographic and healthcare access indicators.

The synchronization patterns identified within the Romanian administrative regions reflect, statistically, temporal similarities between counties’ reported prevalence series. From an interpretative standpoint, these patterns may also suggest that the evolution of Type 2 Diabetes Mellitus prevalence is influenced not only by individual biological risk factors, but also by shared socioeconomic and healthcare-related characteristics. Regions exhibiting a high degree of temporal synchronization may share similar profiles in terms of urbanization level, demographic structure, dietary habits, physical activity patterns, and population access to preventive and curative healthcare services. The differences observed between regions may also reflect variations in healthcare infrastructure and healthcare accessibility. Economically developed and highly urbanized regions generally benefit from a greater capacity for the diagnosis, monitoring, and management of patients with Type 2 Diabetes, whereas predominantly rural areas or regions with more limited healthcare resources may display distinct epidemiological patterns. Furthermore, factors such as population aging, obesity prevalence, and regional lifestyle differences are well-established determinants of Type 2 Diabetes and may contribute to the heterogeneity observed between counties. However, these variables were not included in the present analysis and should be investigated in future studies in order to better understand the mechanisms underlying the identified regional patterns.

These results partially confirm previous observations reported in the scientific literature regarding the existence of spatial–temporal patterns in the regional distribution of diabetes and other chronic diseases, as well as the usefulness of statistical time series analysis for their identification and characterization. Tomov et al. [8] conducted a systematic analysis regarding the applicability of time series analysis in epidemiology, both for forecasting and for identifying regional anomalies. The studies by Zou et al. and Gudziunaite et al. [7,9] emphasize the usefulness of investigating autocorrelations and cross-correlations between time series in order to identify regional shifts (concurrent or delayed).

According to the International Diabetes Federation [24], the distribution of diabetes is not uniform either between or within countries, being influenced by population characteristics, socioeconomic development, and access to healthcare services. Furthermore, European studies have demonstrated that regions characterized by different levels of economic development, urbanization, and healthcare accessibility may display distinct patterns in the prevalence and evolution of Type 2 Diabetes Mellitus [25,26,27]. In the same regard, cross-population comparisons indicate that urbanization and industrialization, associated with specific lifestyle changes such as increased sedentary behavior and consumption of processed food, represent key drivers of variation in Type 2 Diabetes Mellitus prevalence [28,29], a mechanism that may also partly explain the divergent pattern observed in highly urbanized counties such as Cluj in the current dataset.

In Romania, the territorial distribution of diabetes reflects not only demographic characteristics of the population but also regional differences in healthcare accessibility, availability of specialized diabetes centers, healthcare-seeking behavior, and the effectiveness of case detection and monitoring activities [30]. These observations are consistent with findings reported in other European countries, where diabetes prevalence tends to be higher in areas characterized by socioeconomic vulnerability, population aging, and a higher prevalence of metabolic risk factors, particularly obesity and sedentary lifestyles.

Many studies have shown that social determinants of health, including educational attainment, income, occupational status, and access to healthcare services, significantly influence both the risk of developing Type 2 Diabetes Mellitus and the likelihood of timely diagnosis and appropriate disease monitoring. The meta-analysis conducted by Agardh et al. [26] demonstrated that individuals belonging to disadvantaged socioeconomic groups have a significantly higher risk of developing Type 2 Diabetes compared with those from more advantaged groups. Similarly, Espelt et al. [25] reported substantial socioeconomic inequalities in diabetes prevalence across several European countries. In this context, the temporal synchronization observed among certain counties may reflect the presence of similar socioeconomic and demographic characteristics or comparable healthcare delivery models rather than a direct influence between the regions analyzed. Type 2 Diabetes Mellitus is a non-communicable disease, so the identified cross-correlations reflect similarities in temporal evolution of prevalence rates caused by demographic trends and different categories of risk factors, rather than causal relationships between the analyzed regions.

Moreover, recent reports from the World Health Organization [31] and the Organization for Economic Co-operation and Development/European Commission [32] have highlighted that inequalities in access to healthcare contribute to significant differences in early diagnosis, metabolic control, and diabetes case reporting. From this perspective, the regional patterns identified in the present study may reflect local differences in healthcare infrastructure, the availability of diabetes specialists, healthcare accessibility, and the effectiveness of epidemiological surveillance systems.

Therefore, the findings of the present study support the need for region-specific strategies tailored to the demographic, socioeconomic, and organizational characteristics of individual regions, as well as the importance of integrating social determinants of health into the planning of Type 2 Diabetes prevention and control programs. At the same time, they demonstrate the utility of autocorrelation and cross-correlation analyses as complementary tools for identifying spatiotemporal patterns of chronic non-communicable diseases and for characterizing their regional heterogeneity.

From a clinical perspective, the existence of regions with a very high degree of epidemiological synchronization suggests that the risk factors involved in the occurrence and progression of Type 2 Diabetes Mellitus across the Romanian territory act relatively uniformly within these areas. Consequently, prevention, screening, and disease management strategies may be planned at a regional level using common intervention models. In contrast, counties that exhibited atypical patterns or reduced synchronization require further evaluation of local factors, such as access to healthcare services, socio-economic profile, degree of urbanization, obesity prevalence, and lifestyle characteristics [33].

From the perspective of clinical implications and therapeutic strategies, the identification of different temporal patterns in the evolution of Type 2 Diabetes Mellitus prevalence is relevant because it may reflect differences in risk factor control, accessibility of diabetes care services, and the effectiveness of prevention measures implemented at the regional level. This information may support the planning of differentiated screening programs, therapeutic education initiatives, and patient monitoring strategies, with the potential to reduce the severity of diabetes-related complications and the costs associated with healthcare delivery. Similar associations between diabetes burden, healthcare accessibility, and social determinants of health have been extensively documented in the literature [33].

The results obtained have practical relevance for public health authorities because they allow the identification of areas where future pressure on Diabetology services may be similar and predictable. Such information is essential for the efficient planning of the resources required for patient monitoring, prevention of cardiovascular, renal, and neurological complications, and implementation of diabetes education programs. The approach proposed in the present study is actually consistent with the current recommendations emphasizing the importance of population-based strategies and regional adaptation of diabetes care services. Furthermore, the differences observed between regions suggest that Type 2 Diabetes Mellitus prevention and management strategies should not be uniformly implemented at the national level but rather adapted to local epidemiological and socio-demographic characteristics. These findings support the concept that regional variation in diabetes prevalence may reflect broader differences in social determinants of health, healthcare infrastructure, demographic structure, and lifestyle-related factors, although the present ecological analysis does not allow causal inferences to be drawn [33].

*Limitations of the study*: An important limitation of the present study concerns the nature of the data source used. The presented study has an ecological design; the analyses were performed using aggregated county-level data, which did not allow inferences to be made regarding patient-level risk factors, nor causal mechanisms to be identified as explanations for the detected regional patterns. The data analyzed consisted of administratively registered/reported cases of Type 2 Diabetes Mellitus rather than population-based prevalence estimates derived from epidemiological surveys. Since the disease is frequently asymptomatic for long periods, such data sources may underestimate the true burden of disease. Consequently, the identified regional and temporal patterns should be interpreted as reflecting differences in diagnosed and reported disease burden, healthcare-seeking behavior, and surveillance/reporting practices across counties, rather than the true epidemiological prevalence of Type 2 Diabetes Mellitus in the Romanian population. Future studies based on population-representative surveys or, ideally, a national diabetes registry, would allow a more accurate assessment of the real territorial distribution of the disease.

Another possible limitation is a methodological one: the analysis was performed on relatively short time series, comprising only 10 annual observations per county. Although enough for an exploratory analysis of short-term autoregressive structure and inter-county synchronization, this limited series length restricts the reliability of autocorrelation estimates at higher lags and reduces the statistical power to detect more complex temporal patterns, such as cyclical behavior or structural changes. The limited series length also restricted the possibility to check the time series stationarity and to eventually apply detrending procedures, which means that part of the observed autocorrelations and cross-correlations may be the consequence of shared long-term secular trends rather than temporal dependence mechanisms. The uncertainty associated with individual correlation estimates should be taken into account when interpreting the magnitude of the observed coefficients, particularly for correlations calculated at higher lags.

Future studies based on longer surveillance periods, ideally combined with population-based prevalence data, reported globally as well as by comparison according to socio-demographic, economic, or healthcare-related variables (such as urbanization level, household income, population aging, obesity prevalence, etc.) can provide a more robust characterization of the temporal dynamics of Type 2 Diabetes Mellitus in Romania, as well as of the mechanisms responsible for the observed regional heterogeneity. The data source of the current study did not contain information on individual demographic, socioeconomic, behavioral, or clinical characteristics, which meant that adjustments could not be made for potential confounding factors such as age structure, obesity prevalence, educational attainment, income level, urbanization, or healthcare accessibility. In fact, the present analysis was designed only to identify and describe temporal synchronization patterns rather than to determine their underlying causes; the identified synchronization patterns should be interpreted as population-level associations, with limited generalizability, rather than evidence of direct causal relationships.

Regarding *future research directions*, the refinement of time series analysis tools by introducing advanced multivariate analysis models, machine learning techniques, or Bayesian models may allow the integration of additional socio-economic and demographic variables, leading to more robust forecasts and the early detection of emerging epidemiological changes.

## 5. Conclusions

The present study identified distinct temporal and spatial patterns in the evolution of Type 2 Diabetes Mellitus prevalence across Romanian counties, highlighting varying degrees of epidemiological synchronization between the country’s administrative regions. Three major regional patterns were observed: regions characterized by a high degree of synchronization, regions exhibiting mixed synchronization and temporal delays between counties, and regions consisting of a synchronized core accompanied by counties with atypical dynamics. These findings suggest that the burden of Type 2 Diabetes Mellitus is not uniformly distributed across Romania and that important regional differences exist in the temporal evolution of the disease.

Beyond their methodological relevance, the identified patterns may contribute to a better understanding of regional epidemiological dynamics and provide useful insights for public health planning—targeted health strategies, resource allocation policies, and prevention programs adapted to local characteristics. The observed differences between administrative regions support the need for locally adapted prevention and management strategies rather than uniform nationwide approaches. The identification of regions exhibiting similar epidemiological patterns may facilitate more efficient resource allocation and healthcare planning, while counties displaying atypical dynamics may benefit from further investigation of local demographic, socioeconomic, and healthcare-related factors.

However, the findings should be interpreted with caution, given the relatively short observation period, the ecological nature of the study, and the absence of individual-level clinical, demographic, and socioeconomic data. Consequently, the observed temporal and spatial associations should be regarded as exploratory patterns rather than evidence of causal relationships.

Future studies incorporating longer time series, individual-level data, and additional variables related to socioeconomic status, healthcare accessibility, urbanization, population aging, and lifestyle factors may provide a more comprehensive understanding of the mechanisms underlying the regional differences identified in the present analysis. Such approaches may help clarify the determinants of temporal synchronization and regional heterogeneity in Type 2 Diabetes Mellitus prevalence across Romania.

## Figures and Tables

**Table 1:** Summary cross-correlation patterns across all 8 administrative regions of Romania.

Region	Component Counties	Pattern Type	Synchronized Core (Cross-Corr lag 0 > 0.9)	Isolated Counties	Main Lag Structure
North-East (Moldova)	Bacău, Botoșani, Iași, Neamț, Suceava, Vaslui	1—Near-perfect synchronization, no time delays	All 6 counties (>0.95)	-	Lag 0 dominant; lag ±1 (0.669–0.771)
South-East	Brăila, Buzău, Constanța, Galați, Tulcea, Vrancea	2—Homogeneous core + connected heterogeneous subgroup	Buzău, Constanța, Tulcea, Vrancea	Brăila, Galați	Lag 0 + lag ±1 (0.678–0.797)
South-Muntenia	Argeș, Călărași, Dâmbovița, Giurgiu, Ialomița, Prahova, Teleorman	2—Homogeneous core + heterogeneous subgroup (most fragmented pattern)	Argeș, Dâmbovița, Prahova, Teleorman	Ialomița (isolated); Călărași, Giurgiu (partially correlated)	Lag 0 + lag ±1 and ±2 (0.669–0.744)
South-West (Oltenia)	Dolj, Gorj, Mehedinți, Olt, Vâlcea	3—Synchronized core + one isolated county	Dolj, Gorj, Olt, Vâlcea	Mehedinți	Lag 0 + lag ±1 (0.688–0.831)
West (Banat)	Arad, Caraș-Severin, Hunedoara, Timiș	1—Near-perfect synchronization, no time delays	All 4 counties (Arad–Timiș: 0.994)	—	Lag 0 dominant; lag ±1 (0.681–0.786)
North-West	Bihor, Bistrița-Năsăud, Cluj, Maramureș, Satu Mare, Sălaj	3—Synchronized core + one isolated county	Bihor, Bistrița-Năsăud, Maramureș, Satu Mare, Sălaj	Cluj	Lag 0 + lag ±1 (0.688–0.783)
Center	Alba, Brașov, Covasna, Harghita, Mureș, Sibiu	3—Synchronized core + one isolated county	Brașov, Harghita, Sibiu, Covasna	Alba	Lag 0 + lag ±1 (0.670–0.773)
Bucharest-Ilfov	Ilfov, Bucharest	N/A—Strong synchronization	Ilfov–Bucharest (0.862)	—	Lag 0 + lag 1 (0.689)

## Data Availability

The original contributions presented in this study are included in the article. Further inquiries can be directed to the corresponding author.

## Abbreviations

The following abbreviations are used in this manuscript:

CI	Confidence Interval
ARIMA	Autoregressive Integrated Moving Average
LDL	Low-Density Lipoprotein

## Appendix A. The Autocorrelation Coefficients for the Investigated Time Series

	**Lag:** **Autocorrelation Coefficient/*****p*****-Value (Box-Ljung Statistic)**							
	**1**		**2**		**3**		**4**	
Alba	0.119	0.664	0.037	0.901	−0.109	0.938	−0.194	0.884
Arad	0.667	0.015 *	0.382	0.017 *	0.103	0.040 *	−0.102	0.074
Argeș	0.718	0.009 **	0.434	0.008 **	0.147	0.018 *	−0.103	0.036 *
Bacău	0.670	0.014 *	0.374	0.018 *	0.112	0.040 *	−0.076	0.078
Bihor	0.793	0.004 **	0.460	0.003 **	0.125	0.008 **	−0.164	0.015 *
Bistrița-Năsăud	0.644	0.019 *	0.415	0.017 *	0.152	0.037 *	−0.083	0.070
Botoșani	0.704	0.010 *	0.432	0.009 **	0.153	0.020 *	−0.087	0.041 *
Brașov	0.678	0.013 *	0.275	0.027 *	0.067	0.062	−0.089	0.112
Brăila	0.722	0.008 **	0.323	0.014 *	0.032	0.036 *	−0.133	0.064
Buzău	0.717	0.009 **	0.419	0.009 **	0.115	0.021 *	−0.090	0.042 *
Caraș-Severin	0.690	0.012 *	0.359	0.016 *	0.094	0.038 *	−0.089	0.072
Călărași	0.456	0.096	0.248	0.157	0.066	0.287	−0.045	0.432
Cluj	0.186	0.498	0.219	0.554	−0.123	0.696	−0.091	0.807
Constanța	0.745	0.007 **	0.432	0.006 **	0.105	0.016 *	−0.166	0.027 *
Covasna	0.632	0.021 *	0.369	0.025 *	0.212	0.043 *	−0.108	0.078
Dâmbovița	0.677	0.013 *	0.377	0.016 *	0.119	0.037 *	−0.079	0.072
Dolj	0.757	0.006 **	0.442	0.005 **	0.124	0.013 *	−0.147	0.024 *
Galați	0.712	0.009 **	0.271	0.020 *	−0.068	0.047 *	−0.318	0.041 *
Giurgiu	0.449	0.101	0.276	0.147	0.086	0.265	−0.130	0.366
Gorj	0.739	0.007 **	0.433	0.006 **	0.145	0.015 *	−0.102	0.031 *
Harghita	0.718	0.009 **	0.445	0.007 **	0.112	0.018 *	−0.100	0.036 *
Hunedoara	0.665	0.015 *	0.339	0.022 *	0.124	0.048 *	−0.041	0.094
Ialomița	0.188	0.492	−0.121	0.708	0.010	0.875	−0.074	0.938
Iași	0.723	0.008 **	0.426	0.008 **	0.130	0.019 *	−0.111	0.037 *
Ilfov	0.725	0.008 **	0.443	0.007 **	0.152	0.016 *	−0.110	0.032 *
Maramureș	0.708	0.010 *	0.423	0.009 **	0.152	0.021 *	−0.088	0.042 *
Mehedinți	−0.264	0.334	−0.081	0.597	0.127	0.727	0.045	0.853
Mureș	0.437	0.111	0.142	0.241	0.023	0.415	0.020	0.581
Neamț	0.642	0.019 *	0.353	0.025 *	0.075	0.058	−0.025	0.113
Olt	0.747	0.006 **	0.423	0.006 **	0.087	0.016 *	−0.169	0.029 *
Prahova	0.688	0.012 *	0.387	0.014 *	0.125	0.032 *	−0.069	0.063
Satu Mare	0.711	0.009 **	0.417	0.009 **	0.138	0.021 *	−0.084	0.044 *
Salaj	0.657	0.016 *	0.367	0.021 *	0.126	0.045 *	−0.043	0.089
Sibiu	0.717	0.009 **	0.428	0.008 **	0.147	0.019 *	−0.104	0.037 *
Suceava	0.683	0.013 *	0.416	0.012 *	0.145	0.027 *	−0.080	0.054
Teleorman	0.648	0.018 *	0.328	0.027 *	0.090	0.061	−0.056	0.115
Timiș	0.673	0.014 *	0.377	0.017 *	0.082	0.040 *	−0.121	0.072
Tulcea	0.707	0.010 *	0.393	0.011 *	0.099	0.027 *	−0.062	0.055
Vaslui	0.729	0.008 **	0.428	0.007 **	0.144	0.017 *	−0.097	0.035 *
Vâlcea	0.675	0.014 *	0.412	0.014 *	0.167	0.028 *	−0.083	0.056
Vrancea	0.720	0.009 **	0.419	0.008 **	0.143	0.019 *	−0.087	0.040 *
București	0.447	0.103	0.298	0.136	0.139	0.228	−0.168	0.299
**România**	**0.704**	**0.010 ***	**0.415**	**0.010 ***	**0.145**	**0.023 ***	**−0.092**	**0.045 ***

* *p* < 0.05 statistically significant, ** *p* < 0.01 statistically highly significant.

## Appendix B

**Table A1:** The significant cross-correlation coefficients and the corresponding lags: North-East Region.

County	Significant Cross-Correlations
**Bacău-Botoșani**	**Lag 0: 0.989/Lag −1: 0.698/Lag 1: 0.669**
Bacău-Iași	Lag 0: 0.986/Lag −1: 0.673/Lag 1: 0.716
Bacău-Neamț	Lag 0: 0.983
Bacău-Suceava	Lag 0: 0.991/Lag −1: 0.702
Bacău-Vaslui	Lag 0: 0.988/Lag −1: 0.713/Lag 1: 0.673
Botoșani-Iași	Lag 0: 0.991/Lag −1: 0.679/Lag 1: 0.750
Botoșani-Neamț	Lag 0: 0.974/Lag −1: 0.678/Lag 1: 0.670
Botoșani-Suceava	Lag 0: 0.994/Lag −1: 0.714/Lag 1: 0.674
Botoșani-Vaslui	Lag 0: 0.996/Lag −1: 0.726/Lag 1: 0.708
Iași-Neamț	Lag 0: 0.969/Lag −1: 0.720
Iași-Suceava	Lag 0: 0.982/Lag −1: 0.758
Iași-Vaslui	Lag 0: 0.992/Lag −1: 0.771/Lag 1: 0.676
Neamț-Suceava	Lag 0: 0.983/Lag −1: 0.687
Neamț-Vaslui	Lag 0: 0.977/Lag −1: 0.695/Lag 1: 0.682
Suceava-Vaslui	Lag 0: 0.995/Lag −1: 0.694/Lag 1: 0.720

**Table A2:** The significant cross-correlation coefficients and the corresponding lags: South-East Region.

County	Significant Cross-Correlations
Brăila-Buzău	Lag 0: 0.897/Lag 1: 0.713
Brăila-Constanța	Lag 0: 0.835/Lag 1: 0.722
Brăila-Galați	Lag 0: 0.592 not significant
Brăila-Tulcea	Lag 0: 0.919/Lag −1: 0.727/Lag 1: 0.695
Brăila-Vrancea	Lag 0: 0.916/Lag −1: 0.750/Lag 1: 0.678
Buzău-Constanța	Lag 0: 0.981/Lag −1: 0.711/Lag 1: 0.736
Buzău-Galați	Lag 0: 0.824/Lag 1: 0.745
Buzău-Tulcea	Lag 0: 0.988/Lag −1: 0.738/Lag 1: 0.683
Buzău-Vrancea	Lag 0: 0.986/Lag −1: 0.767
Constanța-Galați	Lag 0: 0.890/Lag 1: 0.797
Constanța-Tulcea	Lag 0: 0.943/Lag −1: 0.748
Constanța-Vrancea	Lag 0: 0.954/Lag −1: 0.780
Galați-Tulcea	Lag 0: 0.750/Lag −1: 0.734
Galați-Vrancea	Lag 0: 0.747/Lag −1: 0.737
Tulcea-Vrancea	Lag 0: 0.987/Lag −1: 0.738/Lag 1: 0.696

**Table A3:** The significant cross-correlation coefficients and the corresponding lags: South Region.

County	Significant Cross-Correlations
Argeș-Călărași	Lag 0: 0.872/Lag −1: 0.669
Argeș-Dâmbovița	Lag 0: 0.993/Lag −1: 0.680/Lag 1: 0.708
Argeș-Giurgiu	Lag 0: 0.729/Lag −1: 0.672/Lag −2: 0.729
Argeș-Ialomița	Lag 0: 0.382 not significant
Argeș-Prahova	Lag 0: 0.994/Lag −1: 0.688/Lag 1: 0.715
Argeș-Teleorman	Lag 0: 0.952/Lag −1: 0.735
Călărași-Dâmbovița	Lag 0: 0.880
Călărași-Giurgiu	Lag 0: 0.818
Călărași-Ialomița	Lag 0: 0.613 not significant
Călărași-Prahova	Lag 0: 0.879/Lag 1: 0.678
Călărași-Teleorman	Lag 0: 0.965
Dâmbovița-Giurgiu	Lag 0: 0.689/Lag −2: 0.716
Dâmbovița-Ialomița	Lag 0: 0.355 not significant
Dâmbovița-Prahova	Lag 0: 0.992/Lag −1: 0.673/Lag 1: 0.687
Dâmbovița-Teleorman	Lag 0: 0.952/Lag −1: 0.712
Giurgiu-Ialomița	Lag 0: 0.651
Giurgiu-Prahova	Lag 0: 0.690/Lag 2: 0.735
Giurgiu-Teleorman	Lag 0: 0.805
Ialomița-Prahova	Lag 0: 0.381 not significant
Ialomița-Teleorman	Lag 0: 0.521 not significant
Prahova-Teleorman	Lag 0: 0.955/Lag −1: 0.744

**Table A4:** The significant cross-correlation coefficients and the corresponding lags: South-West Region.

County	Significant Cross-Correlations
Dolj-Gorj	Lag 0: 0.967/Lag −1: 0.831
Dolj-Mehedinți	Lag 0: −0.445 not significant
Dolj-Olt	Lag 0: 0.903/Lag −1: 0.824
Dolj-Vâlcea	Lag 0: 0.965/Lag −1: 0.772
Gorj-Mehedinți	Lag 0: −0.344 not significant
Gorj-Olt	Lag 0: 0.952/Lag −1: 0.744/Lag 1: 0.696
Gorj-Vâlcea	Lag 0: 0.991/Lag −1: 0.688/Lag 1: 0.727
Mehedinți-Olt	Lag 0: −0.315 not significant
Mehedinți-Vâlcea	Lag 0: −0.438 not significant
Olt-Vâlcea	Lag 0: 0.926/Lag 1: 0.721

**Table A5:** The significant cross-correlation coefficients and the corresponding lags: West Region.

County	Significant Cross-Correlations
Arad-Caraș- Severin	Lag 0: 0.937/Lag −1: 0.686
Arad-Hunedoara	Lag 0: 0.937/Lag −1: 0.739
Arad-Timiș	Lag 0: 0.994/Lag 1: 0.685
Caraș- Severin-Hunedoara	Lag 0: 0.949/Lag −1: 0.786
Caraș- Severin-Timiș	Lag 0: 0.939/Lag 1: 0.681
Hunedoara-Timiș	Lag 0: 0.932/Lag 1: 0.741

**Table A6:** The significant cross-correlation coefficients and the corresponding lags: North-West Region.

County	Significant Cross-Correlations
Bihor-Bistrița-Năsăud	Lag 0: 0.936/Lag −1: 0.776
Bihor-Cluj	Lag 0: −0.677
Bihor-Maramureș	Lag 0: 0.973/Lag −1: 0.720/Lag 1: 0.782
Bihor-Satu Mare	Lag 0: 0.969/Lag −1: 0.742/Lag 1: 0.753
Bihor-Sălaj	Lag 0: 0.945/Lag 1: 0.783
Bistrița-Năsăud-Cluj	Lag 0: −0.457 not significant
Bistrița-Năsăud-Maramureș	Lag 0: 0.969/Lag 1: 0.746
Bistrița-Năsăud-Satu Mare	Lag 0: 0.980/Lag 1: 0.736
Bistrița-Năsăud-Sălaj	Lag 0: 0.953/Lag 1: 0.728
Cluj-Maramureș	Lag 0: −0.553 not significant
Cluj-Satu Mare	Lag 0: −0.537 not significant
Cluj-Sălaj	Lag 0: −0.480 not significant
Maramureș-Satu Mare	Lag 0: 0.997/Lag −1: 0.728/Lag 1: 0.688
Maramureș-Sălaj	Lag 0: 0.993/Lag 1: 0.705
Satu Mare-Sălaj	Lag 0: 0.987/Lag 1: 0.722

**Table A7:** The significant cross-correlation coefficients and the corresponding lags: Center Region.

County	Significant Cross-Correlations
Alba-Brașov	Lag 0: 0.412 not significant
Alba-Covasna	Lag 0: 0.724
Alba-Harghita	Lag 0: 0.394 not significant
Alba-Mureș	Lag 0: −0.071 not significant
Alba-Sibiu	Lag 0: 0.295 not significant/Lag 1: 0.709
Brașov-Covasna	Lag 0: 0.807/Lag 1: 0.676
Brașov-Harghita	Lag 0: 0.942/Lag 1: 0.773
Brașov-Mureș	Lag 0: 0.727/Lag 1: 0.706
Brașov-Sibiu	Lag 0: 0.879/Lag 1: 0.768
Covasna-Harghita	Lag 0: 0.920/Lag −1: 0.728
Covasna-Mureș	Lag 0: 0.857
Covasna-Sibiu	Lag 0: 0.962/Lag −1: 0.670/Lag 1: 0.704
Harghita-Mureș	Lag 0: 0.847/Lag 1: 0.687
Harghita-Sibiu	Lag 0: 0.971/Lag 1: 0.767
Mureș-Sibiu	Lag 0: 0.889/Lag −1: 0.668

**Table A8:** The significant cross-correlation coefficients and the corresponding lags: București-Ilfov Region.

County	Significant Cross-Correlations
București city-Ilfov	Lag 0: 0.862/Lag 1: 0.689
